# Optical Coherence Tomography (OCT) Evaluation of Thermal Tissue Alterations After Diode Laser Excision of Oral Leukoplakia (OL)

**DOI:** 10.3390/dj14030168

**Published:** 2026-03-12

**Authors:** Alessio Gambino, Alessandro Magliano, Giorgia El Haddad, Marta Bezzi, Adriana Cafaro, Dora Karimi, Roberto Broccoletti, Paolo Giacomo Arduino

**Affiliations:** 1 Department of Surgical Sciences, CIR Dental School, University of Turin, 10123 Turin, Italy; 2 Department of Mechanical and Aerospace Engineering, Politecnico di Torino, Corso Duca degli Abruzzi 24, 10129 Turin, Italy

**Keywords:** optical coherence tomography, surgical diode laser, oral leukoplakia

## Abstract

**Objectives:** Oral leukoplakia (OL) is the most prevalent oral potentially malignant disorder and requires accurate diagnosis, safe excision, and reliable margin evaluation to minimize recurrence and malignant transformation. Diode laser excision is increasingly adopted due to its precision and favorable clinical outcomes; however, laser-induced thermal effects at surgical margins raise concerns regarding tissue integrity and histopathological reliability. This study aimed to evaluate optical coherence tomography (OCT) as a real-time, high-resolution, non-invasive imaging modality for assessing peri-incisional thermal effects during diode laser excision of non-dysplastic OL. The primary objective was to validate OCT for ultrastructural and morphometric tissue analysis while ensuring preservation of diagnostic readability. **Methods:** A single-center observational case series was conducted at the University of Turin. Thirty patients with clinically and histopathologically confirmed oral leukoplakia without epithelial dysplasia were enrolled and allocated to two groups: 15 lesions excised using a 980 nm diode laser in continuous-wave contact mode (laser group) and 15 lesions removed by conventional scalpel biopsy (control group). Laser excisions were performed with standardized parameters and a circumferential safety margin of 5 mm. Immediately after excision, specimens underwent ex vivo spectral-domain OCT (SD-OCT) imaging to evaluate the epithelial and connective tissue microarchitecture at surgical margins and central lesion areas. OCT acquisition sites were precisely correlated with histological sections. Quantitative OCT measurements of epithelial thickness, lamina propria thickness, and laser-induced thermal alterations were compared with corresponding histological findings. **Results:** OCT consistently provided high-resolution visualization of oral mucosal microarchitecture in both groups, allowing clear identification of epithelial stratification, basement membrane continuity, and lamina propria organization. In the laser group, OCT detected superficial optical alterations at the surgical margins consistent with laser-induced thermal effects, while deeper tissue layers remained structurally readable. Histological analysis revealed mean epithelial and connective tissue thermal alterations of 288.9 μm and 430.3 μm, respectively. OCT-derived measurements showed high concordance with histology, with an overall agreement of 88.5% and no statistically significant differences between OCT and histological assessments. Importantly, laser-induced thermal effects did not impair definitive histopathological diagnosis in any specimen. Comparison with the control group confirmed preserved tissue architecture in scalpel-excised samples and highlighted OCT sensitivity in detecting laser-related structural remodeling. **Conclusions:** OCT proved to be a reliable, non-invasive imaging technique for real-time assessment of diode laser-induced thermal effects during OL excision. The technique accurately delineated tissue microstructure and surgical margins without compromising histopathological interpretation. Integration of OCT into the laser-assisted management of oral potentially malignant disorders may enhance surgical precision, optimize margin control, reduce diagnostic uncertainty, and support individualized follow-up strategies.

## 1. Introduction

Oral potentially malignant disorders (OPMDs) are oral mucosal lesions associated with an increased risk of progression to oral squamous cell carcinoma (OSCC). Among them, oral leukoplakia (OL) is the most prevalent entity encountered in dental practice [1]. The global prevalence of OPMDs is estimated at 4–4.5%, with an overall malignant transformation rate of approximately 8%, although the risk associated with OL is highly variable [2,3]. OL is defined as a predominantly white plaque of questionable malignant potential that cannot be clinically or histopathologically classified as any other definable disease [1]. Non-dysplastic OL is histologically characterized by hyperkeratosis (ortho- or parakeratosis), acanthosis, epithelial atrophy, or their combinations, often associated with a variable chronic inflammatory infiltrate, in the absence of epithelial dysplasia [4]. Despite the lower risk compared with dysplastic lesions, non-dysplastic OL may still undergo malignant transformation, supporting a conservative, risk-based management strategy centered on risk-factor control and long-term follow-up [5]. In recent years, diode laser therapy has gained attention as a minimally invasive approach for managing OPMDs, particularly in cases of OL with a histopathological diagnosis confirming the absence of epithelial dysplasia, where the preservation of healthy tissue and accurate post-treatment monitoring are of paramount importance [6]. At surgical power settings, diode lasers exert photothermal effects, allowing precise tissue ablation with simultaneous coagulation, reduced intraoperative bleeding, and improved visualization of the surgical field [7]. At lower energy densities, diode lasers may induce photobiomodulatory (PBM) effects, modulating inflammation, enhancing cellular metabolism, and accelerating wound healing, thereby improving postoperative patient comfort [8,9]. Despite demonstrated advantages, improper laser settings or excessive thermal exposure can cause carbonization of surgical margins, potentially compromising histopathological assessment and leading to diagnostic inaccuracies [10,11]. However, studies indicate that when excision includes at least 3–5 mm of surrounding healthy tissue, carbonization does not impair diagnostic evaluation and may contribute to reduced lesion recurrence [12,13].

Optical coherence tomography (OCT) is a high-resolution (1–20 μm), non-invasive imaging technique enabling real-time visualization of tissue microarchitecture, effectively serving as an “optical biopsy” without surgical intervention [14,15]. OCT relies on low-coherence interferometry with near-infrared light (770–1300 nm), where backscattered signals generate hyper- and hypo-reflective contrasts to delineate tissue structures [16]. OCT enables visualization of the keratin layer, stratified squamous epithelium, basement membrane, and lamina propria, allowing differentiation between healthy tissue and pathological changes associated with OPMDs [17]. In vivo and ex vivo studies have shown that OCT can detect epithelial thickening and altered layer reflectivity, which correlate with histopathological findings [18]. In OL without dysplasia lesions, OCT reveals irregular epithelial layering and decreased contrast within the lamina propria, highlighting its potential for non-invasive lesion characterization and follow-up [19]. Emerging applications include real-time assessment of carbonization at the margins of excised OL, offering immediate feedback on surgical quality. Its limitations involve limited penetration (1–2 mm) and operator-dependent interpretation, though ongoing advances continue to expand clinical utility [20,21]. This study employed a SD-OCT system device for real-time assessment of peri-incisional thermal effects during laser excisional biopsies of OL, confirming that laser application preserves tissue ultrastructure and diagnostic reliability.

## 2. Materials and Methods

### 2.1. Study Population

This single-center clinical study was conducted at the Department of Surgical Sciences, Oral Medicine Section, CIR Dental School, University of Turin, Italy. All procedures were carried out in accordance with the Declaration of Helsinki [22], as revised in 2000. Written informed consent was obtained from all participants and/or their legal guardians prior to enrollment. The investigation was conducted exclusively on ex vivo human tissue samples obtained from routine surgical procedures performed for standard clinical purposes. All samples were fully anonymized prior to analysis, and no identifiable personal or clinical data were collected or processed. The study complies with the EQUATOR guidelines for research reporting and follows the Standards for Reporting Qualitative Research (SRQR) checklist for the collection, analysis, and reporting of instrumental and clinical data [23]. Inclusion criteria were a clinical and histopathological diagnosis of homogeneous OL without epithelial dysplasia, with a maximum lesion diameter ≤1.5 cm. Written informed consent was obtained from all participants prior to treatment. Exclusion criteria included smoking status, a previous diagnosis of oral carcinoma, Verrucous Proliferative Leukoplakia, OPMDs with dysplasia, as well as the presence of bleeding disorders or ongoing anticoagulant therapy. Smoking was excluded to minimize potential confounding effects on oral mucosal thickness, vascularization, and wound-healing response, which could influence OCT-derived measurements and thermal damage assessment. Eligible patients were allocated to two groups: the laser group and the control group.

### 2.2. Laser Group Protocol

Surgical excision for this group was performed using a 980 nm diode laser (Raffaello Laser by DMT, Lissone, Italy (2 W, continuous-wave, 300 μm fiber, contact mode). Lesions were excised including a circumferential margin of 5 mm from clinically healthy tissue. All surgical procedures were performed by the same operator to minimize inter-operator variability.

### 2.3. Control Group

Control specimens were obtained from peri-lesional, clinically normal mucosa located approximately 3 mm from the lesion margin in patients undergoing scalpel biopsy. This sampling approach avoided any potential laser-induced thermal effects and provided reference tissue representing normal epithelial and lamina propria architecture. OCT scans of these specimens were used as baseline measurements for comparison with the laser group.

All control specimens were imaged immediately after excision under identical ex vivo conditions. The same OCT device, acquisition parameters, and imaging protocol used for the laser-treated specimens were applied to the control group, ensuring methodological consistency and direct comparability between groups.

### 2.4. OCT System and Imaging Acquisition

Ex vivo OCT imaging was performed on excised specimens using a spectral-domain OCT system (Telesto 220, Thorlabs Inc., Newton, NJ, USA) operating at a central wavelength of 1300 nm. The system provided an axial resolution of 5.5 μm, a lateral resolution of 13 μm, a 10 × 10 mm scanning area, and a maximum imaging depth of approximately 3.5 mm. Image optimization was achieved using ThorImageOCT software version 5.4 by adjusting brightness and contrast, with the dynamic range set between 30 and 70 dB to ensure optimal tissue-layer discrimination.

**Figure 1:**
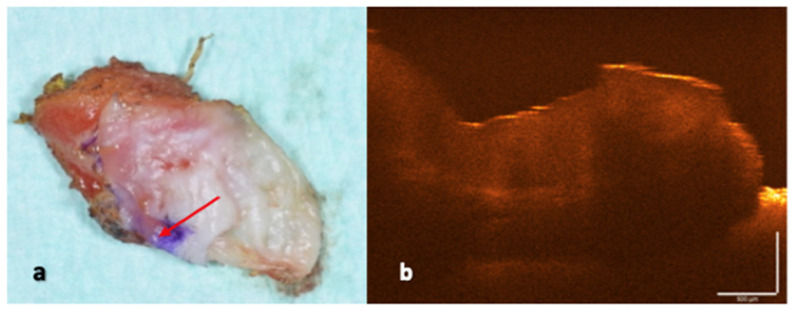
(**a**) Laser-excised specimen with the red arrow indicating the OCT scan site. (**b**) OCT scan of the corresponding surgical margin.

**Figure 2:**
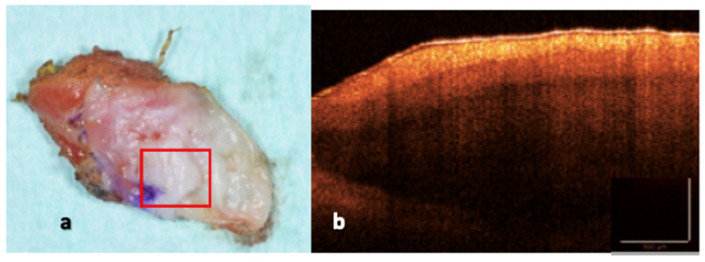
(**a**,**b**) Central specimen OCT scan area unaffected by laser irradiation (red box), used as an internal reference, showing preserved tissue stratification and optical properties of OL.

For each specimen, two standardized OCT acquisitions were obtained: (i) a peripheral OCT scan, acquired at the surgical margin, including peri-lesional tissue to assess pathological features and laser-induced thermal effects (Figure 1), and (ii) a central OCT scan, acquired from an area unaffected by laser irradiation and used as an internal reference (Figure 2).

To ensure precise spatial correlation between OCT findings and histopathological analysis, the OCT-scanned region corresponding to the surgical margin was marked with India ink prior to tissue processing. This procedure enabled accurate identification of the OCT-imaged area by the pathologist and allowed reliable histological measurement of tissue margins corresponding exactly to the OCT acquisition site.

### 2.5. Histological Processing and Quantitative Thermal Damage Assessment

Immediately after OCT imaging, specimens were fixed in 10% neutral-buffered formalin, paraffin-embedded, sectioned, and stained with hematoxylin and eosin (H&E). Histological evaluation was performed under light microscopy, and digital images were acquired at ×100 magnification. Quantitative assessment of laser-induced thermal damage was conducted by a blinded pathologist using dedicated digital analysis software. Thermal alteration was measured in microns from the surgical margin toward histologically readable tissue. The extent of thermal damage was defined as the mean maximum unreadable area observed in both epithelial and connective tissue compartments. Connective tissue alterations were characterized by basophilic changes consistent with hemocoagulative phenomena.

### 2.6. OCT-Based Thermal Damage Quantification and Histology Correlation

**Figure 3:**
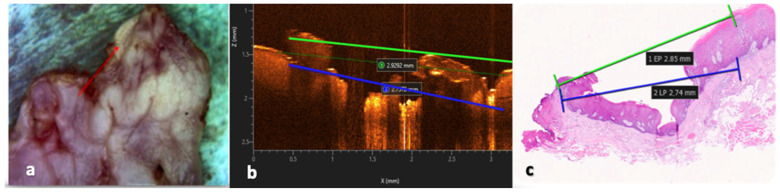
OCT–histology correlation of laser-induced thermal damage: (**a**) peripheral OCT scan at the surgical margin (red line); (**b**) OCT-based measurement of epithelial thermal alteration from surface disruption to restored reflectivity (green line) and connective tissue hyper-reflective areas corresponding to hypercoagulated vessels (blue line); (**c**) histological measurement of thermal damage defined as the maximum distance between epithelial carbonization (green line) and collagen denaturation in the connective tissue (blue line).

Histological measurements were directly correlated with the corresponding OCT images to assess laser-induced thermal damage at the surgical margins. In OCT scans, thermal damage was operationally defined as the presence of a superficial optically unreadable or highly scattered zones, characterized by loss of normal epithelial–connective tissue stratification, increased backscattering intensity, and attenuation of signal penetration depth. Quantitative OCT measurements were performed by calculating, in microns, the vertical thickness of this altered optical layer, measured from the tissue surface to the point at which normal tissue architecture and signal readability were restored. Measurements were obtained on peripheral OCT scans at predefined locations along the surgical margin and averaged to reduce local variability. All OCT measurements were performed by a single trained examiner to ensure methodological consistency and reduce inter-operator variability. OCT-derived thermal damage measurements were then compared with histological findings, including epithelial surface carbonization, collagen denaturation within the connective tissue, and vascular alterations, to validate the correspondence between optical and microscopic indicators of laser-induced thermal effects (Figure 3).

### 2.7. Statistical Analysis

Agreement between OCT-derived and histologically measured tissue thickness was assessed using the intraclass correlation coefficient (ICC), which evaluates both the consistency and absolute agreement between two measurement methods. In addition, paired comparisons were performed using the Wilcoxon signed-rank test for non-normally distributed differences to confirm the absence of significant systematic bias.

## 3. Results

### 3.1. Sample Characteristics

Thirty specimens histopathologically diagnosed as oral leukoplakia without epithelial dysplasia were analyzed (17 males, 13 females; mean age: 49.35 years). Fifteen lesions were excised using a diode laser (laser group), and fifteen were removed with a scalpel (control group).

### 3.2. OCT Assessment

**Figure 4:**
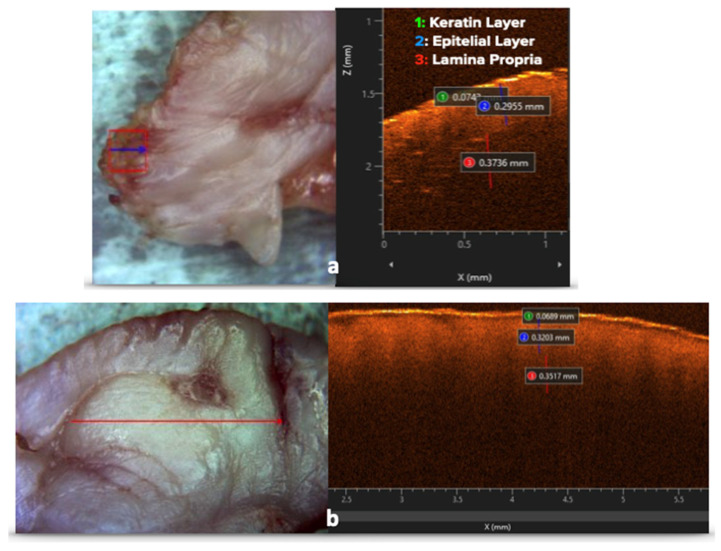
OCT measurements allowed identification of the pathological pattern of oral leukoplakia even in the most peripheral scans at the surgical margin (**a**); comparison with central scans (**b**) showed no significant differences in tissue architecture.

OCT imaging identified the pathological pattern of oral leukoplakia in all samples, including peripheral scans at the surgical margins, where peri-lesional healthy tissue was clearly distinguishable (Figure 4). No significant differences were detected between peripheral and central OCT scans in epithelial or connective tissue thickness (Table 1), indicating that diode laser-induced thermal effects did not compromise the diagnostic assessment of lesion margins.

### 3.3. Histological Thermal Damage

Histological analysis revealed consistent laser-induced thermal alterations, including superficial epithelial carbonization and collagen denaturation within the connective tissue, associated with eosinophilia and vascular changes. These features were clearly measurable in all specimens.

### 3.4. OCT–Histology Quantitative Comparison

Thermal damage in OCT was quantified by measuring epithelial surface disruption up to restored reflectivity and localized hyper-reflective areas in the connective tissue corresponding to hypercoagulated vessels. Measurements from OCT and histology were compared using a standard error of 0.05 mm.

The mean epithelial thermal damage measured histologically was 288.9 µm, while the mean connective tissue damage was 430.3 µm. OCT analysis showed mean epithelial remodeling of 0.028 ± 0.05 mm and connective tissue remodeling of 0.040 ± 0.05 mm. The overall concordance between OCT and histological measurements was 88.5%. A definitive histopathological diagnosis was obtained in all cases.

### 3.5. OCT–Histology Agreement Analysis 

The OCT and histological measurements of epithelial and lamina propria thickness for all samples are summarized in Table 1 and Table 2. The mean epithelial thickness measured by OCT was 0.414 ± 0.163 mm, compared with 0.470 ± 0.185 mm with histology. The mean lamina propria thickness was 0.507 ± 0.416 mm with OCT and 0.627 ± 0.509 mm with histology. OCT accurately identified the pathological pattern of leukoplakia, including the peripheral margins. Statistical analysis confirmed good agreement between the OCT and histological measurements, with an ICC of 0.88 (95% CI: 0.81–0.93) and no significant differences detected by the Wilcoxon signed-rank test for either epithelial (*p* = 0.42) or lamina propria (*p* = 0.36) compartments, supporting the reliability of OCT for margin assessment (Table 3).

### 3.6. Comparison of Laser Group with Control Group

OCT measurements of laser-treated OL were compared with clinically normal peri-lesional mucosa obtained from scalpel biopsy, serving as a healthy control group (Table 4).

The mean epithelium thickness in laser-treated tissue was 0.414 ± 0.163 mm (median: 0.40 mm; range: 0.21–0.80 mm), while the healthy control epithelium measured 0.276 ± 0.050 mm (median: 0.27 mm; range: 0.19–0.36 mm). The lamina propria thickness was 0.507 ± 0.416 mm (median: 0.40 mm; range: 0.18–1.50 mm) in laser-treated tissue and 0.443 ± 0.089 mm (median: 0.44 mm; range: 0.28–0.55 mm) in healthy controls. A Mann–Whitney U test confirmed significant differences between laser-treated and healthy tissue for both the epithelium (*p* = 0.021) and lamina propria (*p* = 0.034).

## 4. Discussion

This study evaluated the structural and histological effects of diode laser in OL without epithelial dysplasia excision by integrating OCT imaging with conventional histological analysis.

OCT imaging consistently identified characteristic patterns of OL in all specimens, including peripheral scans at surgical margins. Peripheral regions, at approximately 3 mm from the lesion edge, clearly showed clinically normal mucosa, indicating that diode laser-induced thermal effects did not obscure the structural assessment of margins. The absence of significant differences between central and peripheral OCT measurements in epithelial or connective tissue thickness supports the reliability of OCT for margin evaluation following laser excision, aligning with prior observations that OCT can non-invasively delineate mucosal microarchitecture in precancerous conditions [24,25].

Quantitative comparison with histology revealed high concordance (88.5%), confirming that OCT-derived measurements reliably reflect histological architecture and thermal effects. OCT was able to detect epithelial surface disruption and changes in connective tissue reflectivity corresponding to localized thermal alteration, whereas histology demonstrated superficial epithelial carbonization and collagen denaturation with eosinophilic vascular changes. Importantly, these thermal alterations did not prevent definitive histological diagnosis in any case, supporting the notion that appropriately parameterized laser excision produces limited and predictable effects that do not compromise pathological interpretation [26].

Comparison with healthy control tissue further emphasized OCT’s performance in identifying laser-related structural modifications. Both epithelial and lamina propria thickness were significantly greater in laser-treated sites compared to the control group. These results illustrate OCT’s ability to quantify subtle architectural changes following laser exposure, with control reference measurements obtained from clinically normal mucosa sufficiently distant from the lesion to minimize thermal artifacts [27,28]. Larger human OCT studies have reported high diagnostic accuracy for OL and other oral mucosal disorders when directly compared with biopsy results, with a sensitivity and specificity approaching 98% or greater for OL in site-registered OCT protocols [29]. From a clinical perspective, these findings suggest that OCT may provide useful adjunctive information in the management of OPMDs. Its non-invasive visualization of tissue microstructure allows near-real-time assessment of excision margins and laser-induced changes; however, further in vivo validation studies are required to confirm its clinical applicability [30,31].

Histological studies and systematic reviews have shown that despite the presence of peripheral thermal damage with various laser wavelengths this effect does not hinder histopathological diagnosis of oral soft tissue biopsies when appropriate parameters and margins are used, supporting the safety and practical applicability of laser excision in routine clinical practice [32]. Nevertheless, limitations of this study include the modest sample size and the ex vivo nature of OCT imaging. Future research should focus on in vivo OCT evaluation, larger multi-center cohorts, and comparative studies across different laser wavelengths and parameters to further optimize surgical protocols for OPMDs. The integration of OCT into surgical workflows may offer additional support for margin evaluation and structural assessment, but its routine clinical implementation requires further validation and standardized acquisition protocols. Recent advances integrating artificial intelligence with OCT imaging have shown promising improvements in diagnostic performance, including a high sensitivity and specificity; however, these approaches remain investigational and require further validation before routine clinical adoption [29]. Although this study was limited to OL without epithelial dysplasia, the present findings have potential relevance for dysplastic lesions. Appropriate diode laser excision with adequate margins (≥3–5 mm) has been shown not to compromise histopathological assessment and may therefore be considered clinically feasible in dysplastic OL, where accurate grading and clear margins are critical for risk stratification and management. Additionally, OCT has demonstrated high diagnostic performance in differentiating grades of oral epithelial dysplasia and assessing lesion margins, with a sensitivity and specificity exceeding 80% across dysplasia severities and substantial agreement with histopathology, supporting its role as a non-invasive adjunctive tool in lesion characterization and margin evaluation. However, OCT cannot replace histopathological biopsy for definitive dysplasia grading, and specific clinical studies focusing on dysplastic OL are still required to confirm the generalizability of these results to this higher-risk subgroup [33].

Although the present study used an ex vivo OCT protocol to ensure precise correlation with histopathology, the technical features of the spectral-domain OCT system (real-time acquisition and high resolution) make intraoperative application feasible. The ex vivo design was chosen to standardize imaging conditions and avoid motion artifacts; however, the acquisition protocol and interpretation criteria can be directly translated to an intraoperative setting, where OCT could assist in real-time margin assessment [34,35].

## 5. Conclusions

This study confirms that OCT is a reliable, non-invasive tool for the evaluation of OL excised with a diode laser. OCT consistently delineated microstructural features of OL and provided a clear visualization of surgical margins, demonstrating that laser-induced thermal effects do not compromise image interpretation or histopathological diagnosis. A high level of concordance between OCT and histology was observed for both epithelial and lamina propria thickness, supporting OCT’s accuracy in detecting and quantifying laser-related tissue alterations. Comparison with healthy mucosa further highlighted OCT’s sensitivity in identifying subtle structural changes. These findings suggest that OCT may provide valuable adjunctive information for intraoperative margin assessment and postoperative monitoring in OL management; however, its clinical implementation requires further in vivo validation. Future studies on larger cohorts are warranted to confirm these results and to optimize laser-assisted surgical protocols under standardized conditions.

## Figures and Tables

**Table 1:** OCT and histological measurements of epithelial thickness of the epithelial compartment obtained by OCT and corresponding histological analysis.

Patient Number	OCT Epithelium (mm)	Histology Epithelium (mm)
1	0.40	0.83
2	0.34	0.29
3	0.62	0.56
4	0.43	0.39
5	0.24	0.25
6	0.30	0.34
7	0.60	0.75
8	0.63	0.68
9	0.49	0.51
10	0.43	0.44
11	0.22	0.24
12	0.21	0.25
13	0.80	0.85
14	0.25	0.28
15	0.31	0.35

**Table 2:** OCT and histological measurements of lamina propria thickness obtained by OCT and corresponding histological analysis.

Patient Number	OCT Lamina Propria (mm)	Histology Lamina Propria (mm)
1	0.22	0.55
2	0.24	0.30
3	0.40	0.45
4	0.46	0.51
5	0.28	0.35
6	0.18	0.20
7	0.55	1.10
8	1.50	1.90
9	0.55	0.58
10	0.42	0.46
11	0.36	0.39
12	0.76	0.80
13	0.78	0.69
14	0.25	0.30
15	0.36	0.40

**Table 3:** Descriptive statistics of epithelial and lamina propria thickness measured by OCT and corresponding histological values in laser-treated OL.

Tissue Compartment	Measurement Method	Mean ± SD (mm)	Median (mm)	Range (mm)
Epithelium	OCT	0.41 ± 0.16	0.40	0.21–0.80
Epithelium	Histology	0.47 ± 0.19	0.39	0.24–0.85
Lamina propria	OCT	0.51 ± 0.42	0.40	0.18–1.50
Lamina propria	Histology	0.63 ± 0.51	0.46	0.20–1.90

**Table 4:** Laser-treated vs. healthy control tissue (OCT).

Tissue Compartment	Group	Mean ± SD (mm)	Median (mm)	Range (mm)	95% CI (mm)	*p*-Value
Epithelium	Laser Group	0.414 ± 0.163	0.40	0.21–0.80	0.226–0.376	0.021 *
Epithelium	Control Group	0.276 ± 0.050	0.27	0.19–0.36	–	–
Lamina Propria	Laser Group	0.507 ± 0.416	0.40	0.18–1.50	0.295–0.621	0.034 *
Lamina Propria	Control Group	0.443 ± 0.089	0.44	0.28–0.55	–	-

* *p* < 0.05, Mann–Whitney U test.

## Data Availability

Data are available from the corresponding author upon request due to privacy and ethical restrictions.

## Abbreviations

The following abbreviations are used in this manuscript:

OCT	Optical Coherence Tomography
OPMDs	Oral Potentially Malignant Disorders
OL	Oral Leukoplakia
